# Evaluating the predictive value of angiogenesis-related genes for prognosis and immunotherapy response in prostate adenocarcinoma using machine learning and experimental approaches

**DOI:** 10.3389/fimmu.2024.1416914

**Published:** 2024-05-16

**Authors:** YaXuan Wang, JiaXing He, QingYun Zhao, Ji Bo, Yu Zhou, HaoDong Sun, BeiChen Ding, MingHua Ren

**Affiliations:** Department of Urology, The First Affiliated Hospital of Harbin Medical University, Harbin, China

**Keywords:** prognosis, angiogenesis, machine learning, PRAD, biomarker

## Abstract

**Background:**

Angiogenesis, the process of forming new blood vessels from pre-existing ones, plays a crucial role in the development and advancement of cancer. Although blocking angiogenesis has shown success in treating different types of solid tumors, its relevance in prostate adenocarcinoma (PRAD) has not been thoroughly investigated.

**Method:**

This study utilized the WGCNA method to identify angiogenesis-related genes and assessed their diagnostic and prognostic value in patients with PRAD through cluster analysis. A diagnostic model was constructed using multiple machine learning techniques, while a prognostic model was developed employing the LASSO algorithm, underscoring the relevance of angiogenesis-related genes in PRAD. Further analysis identified MAP7D3 as the most significant prognostic gene among angiogenesis-related genes using multivariate Cox regression analysis and various machine learning algorithms. The study also investigated the correlation between MAP7D3 and immune infiltration as well as drug sensitivity in PRAD. Molecular docking analysis was conducted to assess the binding affinity of MAP7D3 to angiogenic drugs. Immunohistochemistry analysis of 60 PRAD tissue samples confirmed the expression and prognostic value of MAP7D3.

**Result:**

Overall, the study identified 10 key angiogenesis-related genes through WGCNA and demonstrated their potential prognostic and immune-related implications in PRAD patients. MAP7D3 is found to be closely associated with the prognosis of PRAD and its response to immunotherapy. Through molecular docking studies, it was revealed that MAP7D3 exhibits a high binding affinity to angiogenic drugs. Furthermore, experimental data confirmed the upregulation of MAP7D3 in PRAD, correlating with a poorer prognosis.

**Conclusion:**

Our study confirmed the important role of angiogenesis-related genes in PRAD and identified a new angiogenesis-related target MAP7D3.

## 1 Introduction

Prostate adenocarcinoma (PRAD) is the most common solid tumor and the fifth leading cause of cancer death in men, and is now considered a global public health problem (1). Various genetic and environmental factors, including advanced age and family history of PRAD, have been identified as risk factors (2). The majority of patients present with nonspecific symptoms such as decreased urinary flow, urgency, increased nocturia, and incomplete bladder emptying, leading to late-stage diagnosis and high mortality rates (3). While advancements in radiotherapy, targeted therapy, and immunotherapy have improved patient outcomes, the challenge of achieving a complete cure for PRAD patients remains significant. Understanding the etiology and pathogenesis of PRAD and developing new treatment strategies are crucial in addressing this pressing issue.

Angiogenesis is the process of developing new vascular structures from existing capillaries or post-capillary venules. This involves the degradation of the vascular basement membrane, stimulation, proliferation, and migration of vascular endothelial cells, and remodeling to form new blood vessels and networks (4). Hypoxia, particularly within the hypoxic regions of solid tumors, is a key factor influencing tumor cell response by impeding the infiltration of immune cells and reducing their anti-tumor activity (5). The response of tumor endothelial cells to hypoxic signals acts as a switch for angiogenesis (6). Disruption of angiogenesis through anti-angiogenic therapies can result in significant hypoxia and promote resistance to tumor drugs (7). Tumors require new blood vessels to support their growth by supplying oxygen and nutrients and eliminating metabolic waste. Angiogenesis is typically initiated once a tumor reaches a certain size, typically around 1–2 mm in diameter (8). Solid malignant tumors, such as PRAD, rely on a sufficient blood supply to support their growth, development, and spread (9). Recent studies have confirmed the role of exosomal PGAM1 in promoting PRAD angiogenesis, suggesting its potential as a diagnostic marker for PRAD metastasis (10). Interleukin-30 disrupts prostate cancer cross-talk with endothelial cells by enhancing angiogenesis (11). The expression of FOXA1 in prostate cancer is positively associated with cancer vessel lymphatic invasion and metastasis, likely due to its regulation of angiogenesis (12). Additionally, Ephrin-A2 has been found to promote prostate cancer metastasis by stimulating angiogenesis (13). Therapy targeting angiogenesis not only inhibits the growth of tumor blood vessels but also restores their abnormal structure within tumors. This normalization of the vasculature shifts suppressive immune conditions to an immune-stimulated state. The activation of the immune system due to therapy also aids in improving the structure of blood vessels, creating a beneficial cycle of mutual enhancement (14). Therefore, a thorough investigation into the role of angiogenesis in PRAD not only aids in early detection but also holds significant value for immunotherapy in PRAD.

The purpose of this study was to explore the importance of angiogenic genes in the diagnosis, prognosis, and treatment outcome of PRAD. Initially, the ssGSEA algorithm was utilized to assess angiogenesis scores in 498 samples from the TCGA-PRAD dataset (15, 16). Subsequently, 10 prognostic differential genes related to angiogenesis in PRAD were identified using the weighted gene co-expression network analysis (WGCNA) method. Cluster analysis was then conducted based on the expression of these 10 genes to evaluate their correlation with patient prognosis, response to immunotherapy and chemotherapy. An angiogenesis-related diagnostic model was developed using 60 algorithms on the TCGA-PRAD dataset and validated with the GSE62872 dataset. Additionally, a prognostic model focusing on angiogenesis was constructed using the least absolute shrinkage and selection operator (LASSO) algorithm, demonstrating high predictive accuracy for PRAD patient outcomes. Furthermore, the most significant angiogenesis-related prognostic gene, MAP7D3, was identified in PRAD using three machine learning methods. The study has established a close relationship between MAP7D3 and immunotherapy and chemotherapy in patients with PRAD. Additionally, the high correlation between MAP7D3 and angiogenesis-targeting drugs was confirmed using molecular docking methods, suggesting a potential role for MAP7D3 in angiogenesis-targeting therapy.

## 2 Materials and methods

### 2.1 Data acquisition

There are 498 PRAD samples and 52 corresponding normal samples from the TCGA database included in the study. Additionally, our study also includes 264 PRAD samples and 160 normal prostate samples from the GSE62872 dataset. Immunoinfiltration analysis of MAP7D3 in PRAD was conducted using the GSE143791 dataset from the TISCH website. Additionally, 60 cases of PRAD tissue and paired para-cancerous tissue were procured from Shanghai Outdo Biotech Company. The patients included in the tissue chip study underwent surgery between January 2011 and December 2014, with a follow-up period extending from November 2021, spanning 6 to 10 years.

### 2.2 Consistency cluster analysis

To analyze consistency, we utilized the ConsensusClusterPlus R package (v1.54.0) (17). A total of 100 samples, each comprising 80%, were drawn repeatedly, resulting in the generation of up to 6 clusters. The hierarchical clustering approach involved setting clusterAlg=“hc” and innerLinkage=‘ward.D2’.

### 2.3 Constructing diagnostic and prognostic models

We utilized multiple machine learning algorithms and developed 108 combinations of different algorithms to build PRAD diagnostic models. The training set consisted of the TCGA-PRAD dataset, while GSE62872 served as the verification set. For each algorithm combination, we computed the AUC value, and the combination with the highest average AUC was deemed the most optimal (18). The prognostic model was characterized by LASSO regression algorithm and 10-fold cross-validation was used for this analysis (19, 20). The R software glmnet package was used for this analysis.

### 2.4 Immune infiltration and chemotherapeutic drug sensitivity analysis

In order to assess the immune scores of genes related to angiogenesis in PRAD, we utilized the immunedeconv tool (21). For our study, we specifically employed the xCell algorithm due to its ability to evaluate a wide range of immune cell types, making it well-suited for our investigation. Furthermore, we utilized the Genomics Database for Cancer Drug Sensitivity (GDSC) to predict the response to chemotherapy for each sample. This prediction process was conducted using the pRRophetic R package. The tumor immunophenotyping (TIP) method complements the existing ssGSEA and CIBERSORT methods and can systematically track and analyze the proportion of tumor-infiltrating immune cells in the tumor immune cycle (22). Our study utilized the TIP method to investigate the relationship between angiogenesis-related genes and immune cell infiltration in PRAD. Furthermore, we employed the TISCH2 database to assess the correlation between MAP7D3 and immune cell infiltration in PRAD (23).

### 2.5 Gene Set Enrichment Analysis

In Gene Set Enrichment Analysis (GSEA), we utilized version 3.0 of the GSEA software (24). In the content section of cluster analysis, we grouped the data by cluster 1 and cluster 2. For enrichment analysis on MAP7D3, we utilized the median expression of MAP7D3 as the threshold. Samples with expression levels higher than the median were categorized as the high-expression group, while samples with expression levels lower than the median were categorized as the low-expression group. Then gene sets corresponding to relevant signaling pathways are extracted from the molecular feature database, and the signaling pathways and molecular mechanisms related to gene expression are analyzed (25). The genome sizes were constrained between 5 and 5000, with one thousand resamplings conducted. A statistically significant P value below 0.05 was considered for result interpretation.

### 2.6 Correlation analysis of MAP7D3 with angiogenesis-targeting drugs

To assess the binding affinity of the key gene MAP7D3 with angiogenic drugs, we employed a molecular docking approach for analysis. The CB-Dock2 website (26) was utilized as a valuable tool in our study, utilizing Vina score to assess the binding affinity of genes and drugs. A Vina score below 5.0 kcal/mol is commonly considered indicative of a more robust binding interaction between the gene and drug.

### 2.7 Immunohistochemical staining analysis of MAP7D3 expression in PRAD tissues

The PRAD tissue chip underwent a series of preparation steps including heating in an oven at 85°C for 15 minutes, soaking in xylene for 20 minutes, immersion in various concentrations of ethanol, citric acid treatment with antigen retrieval in a pressure cooker, and subsequent rinsing with PBS and hydrogen peroxide solution. The chip was then incubated with MAP7D3 antibody (bs-18668R) overnight at 4°C, followed by rinsing, incubation with secondary antibodies, DAB reagent treatment for color development, and staining with hematoxylin. Immunostaining intensity was scored from 0 to 3 based on reaction strength, and a scale from 1 to 4 was used to assess the proportion of positive staining. The final expression score was calculated by multiplying the intensity and scale scores, with scores ranging from 0 to 5 indicating low expression and scores from 6 to 12 indicating high expression.

### 2.8 Statistical analysis

All the analysis methods and R package were implemented by R version 4.0.3. The statistical difference of two groups was compared through the Wilcox test. A statistically significant difference is indicated by p < 0.05.

## 3 Results

### 3.1 Identification of angiogenesis-related genes in PRAD

**Figure 1:**
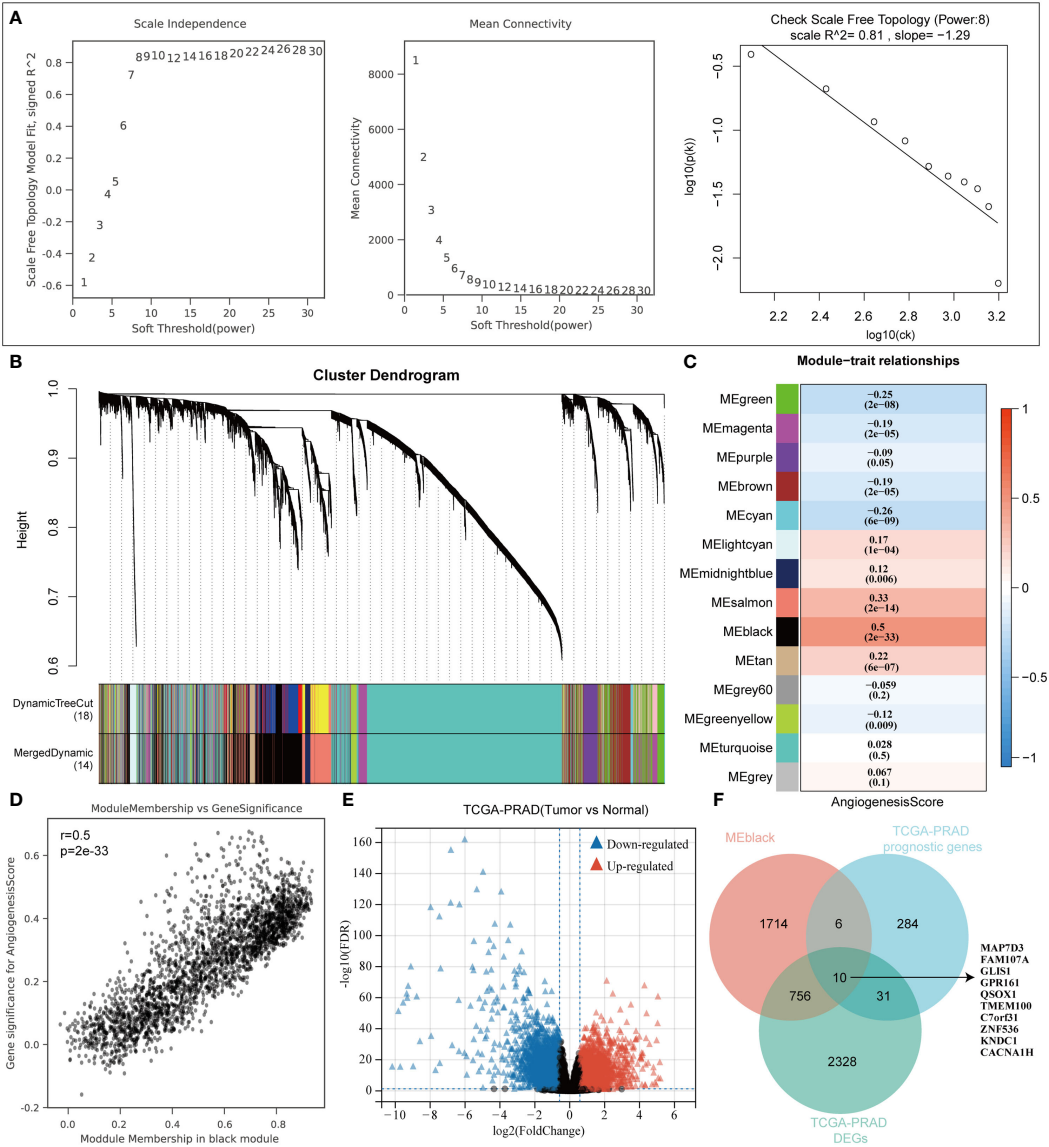
WGCNA algorithm screens angiogenesis-related genes. **(A)** WGCNA Network Construction Parameters. **(B)** The upper part of the figure shows the gene clustering tree constructed on the weighted correlation coefficients, and the lower part of the figure is divided into the distribution of genes in each module. **(C)** Heatmap of trait module associations. **(D)** Scatterplot of Angiogenesis and Module Gene Association. **(E)** TCGA-PRAD dataset variance analysis volcano plot. **(F)** Venn diagrams to map angiogenic prognostic differential genes.

Within the TCGA-PRAD dataset, 498 samples were analyzed to compute the angiogenesis score for each using the ssGSEA approach. Subsequently, the samples were segregated into two categories depending on the median angiogenesis score. In the analysis conducted, the parameter power for the weight of the adjacency matrix was set to 8 to guarantee a scale-free distribution of the network. WGCNA, a computational method utilized for deriving module information from extensive expression data, characterizes a module as a cluster of genes exhibiting comparable expression profiles (**Figure 1A**). Pearson correlation analysis was then performed to assess the correlation between module characteristic genes and traits (**Figures 1B, C**). Notably, the black module exhibited the highest correlation (correlation coefficient of 0.5) with angiogenesis (**Figure 1D**). Differential analysis of the TCGA-PRAD data set between cancer and normal tissues identified 3125 differential genes (**Figure 1E**). P < 0.05 and Log2 (Fold Change) >1.3 or Log2 (Fold Change) < -1.3 were defined as thresholds for differential expression screening. Subsequently, prognostic gene analysis in the TCGA-PRAD data set revealed 331 prognostic genes. By overlaying these sets using a Venn diagram, we identified 10 prognostic-related angiogenesis differential genes (**Figure 1F**).

### 3.2 Consensus clustering analysis of angiogenesis regulatory factors

**Figure 2:**
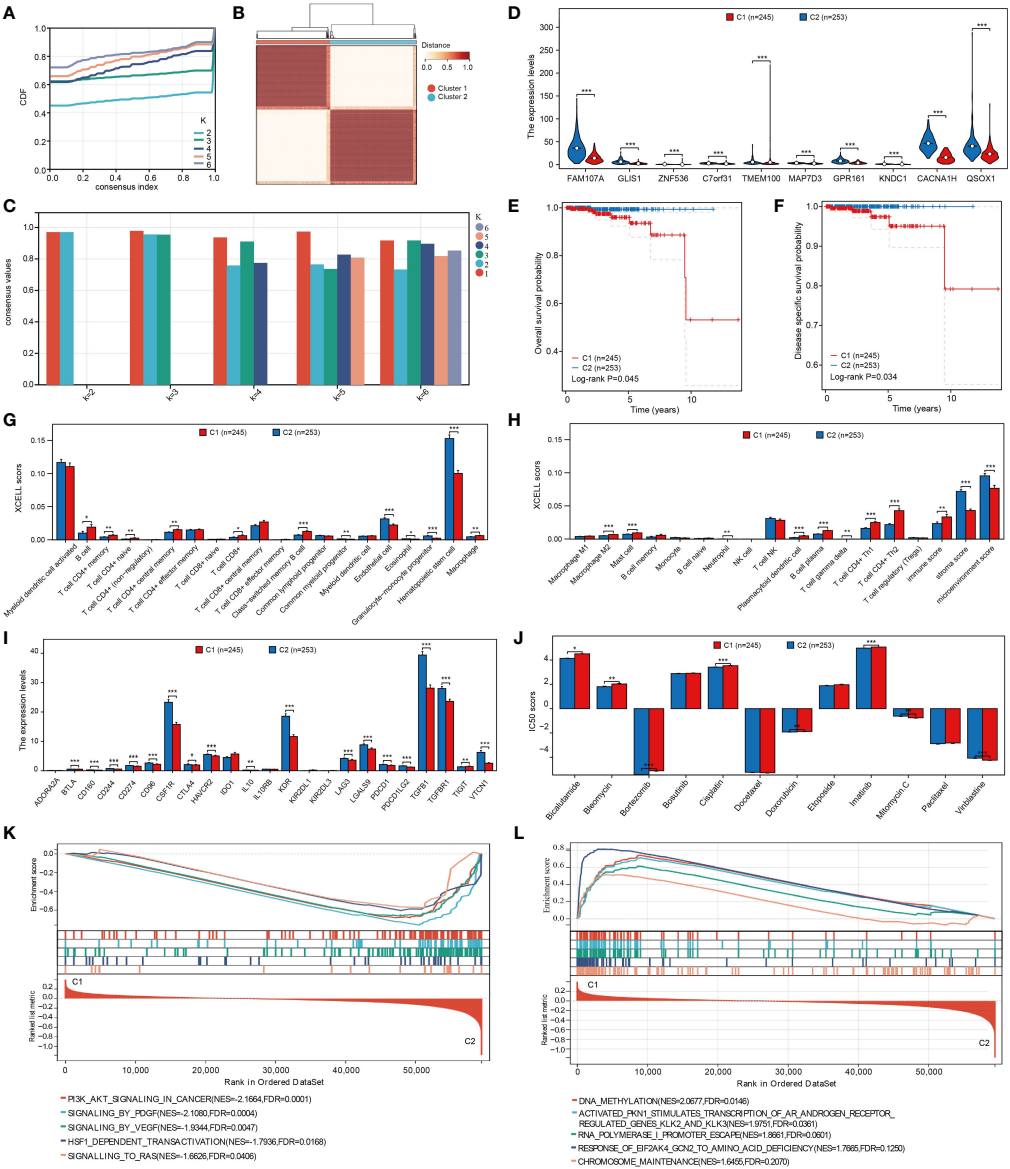
Cluster analysis of PRAD patients based on angiogenesis genes. **(A)** Cumulative distribution curve. **(B)** Clustering heatmap. **(C)** Evaluation of average consistency within clustered groups. **(D)** Differential expression of angiogenesis-related genes in clusters. **(E, F)** Differences in overall and disease- specific survival between clusters. **(G, H)** Analysis of different levels of immune cell infiltration between clusters. **(I)** Analysis of expression levels of different immunoinhibitors among clusters. **(J)** Analysis of differences in IC50 scores of different chemotherapeutic agents between clusters. **(K, L)** Analysis of gene enrichment between clusters. *p < 0.05, **p < 0.01 and ***p < 0.001.

The optimal cluster stability for k = 2 was determined by assessing the similarity in expression levels of angiogenic regulatory factors and fuzzy clustering measures (k = 2 to 6). A total of 499 PRAD patients were then classified into two clusters: cluster 1 (n = 245) and cluster 2 (n = 253), based on their expression levels of angiogenic regulatory factors (**Figures 2A, B**). In addition, according to the average consistency evaluation within the clustering group, here, the number of clusters with the highest average consistency within the group is also K=2 (**Figure 2C**). Subsequently, we assessed the expression variances of angiogenesis-related prognostic differential genes including MAP7D3, FAM107A, GLIS1, GPR161, QSOX1, TMEM100, C7orf31, ZNF536, KNDC1, and CACNA1H in the two clusters. Our analysis revealed significant differences in all of the mentioned genes (**Figure 2D**). Patients in cluster 1 exhibited a worse prognosis in terms of both overall survival and disease-specific survival among PRAD patients (**Figures 2E, F**). Given this conclusion, we are interested in understanding the regulatory mechanism. The immune microenvironment plays a crucial role in tumor progression, with angiogenesis closely linked to immune mechanisms in tumors. Therefore, we hypothesize that the difference in prognosis between the two patient clusters may be related to immune mechanisms. This study delved deeper into the relationship between two distinct clusters and immune cell infiltration in patients with prostate adenocarcinoma (PRAD). By utilizing the xCell algorithm, we assessed levels of immune cell infiltration in 38 cells and identified significant differences in 23 cells between the two clusters (**Figures 2G, H**). This suggests that the varying patient prognoses in these clusters may be associated with differences in immune cell infiltration. As a novel form of tumor immunotherapy drug, immune checkpoint inhibitors are crucial in the field of tumor immunotherapy (27). Our study focused on analyzing the variations in the expression of immunosuppressants across different clusters. Out of the 23 immunoinhibitors studied, 18 displayed significant differences between the clusters (**Figure 2I**). Furthermore, we analyzed the IC50 scores of commonly used clinical chemotherapy drugs in the two clusters, uncovering significant differences in the IC50 scores of 8 chemotherapy drugs (**Figure 2J**). In order to deeply analyze the underlying mechanisms of the above results, we also performed gene enrichment analysis on the 2 clusters, and we found that cluster 2 was significantly associated with PI3K/AKT, PDGF, VEGF, and RAS signaling pathways, whereas cluster 1 was associated with factors such as DNA methylation (**Figures 2K, L**).

### 3.3 Construction of diagnostic models

**Figure 3:**
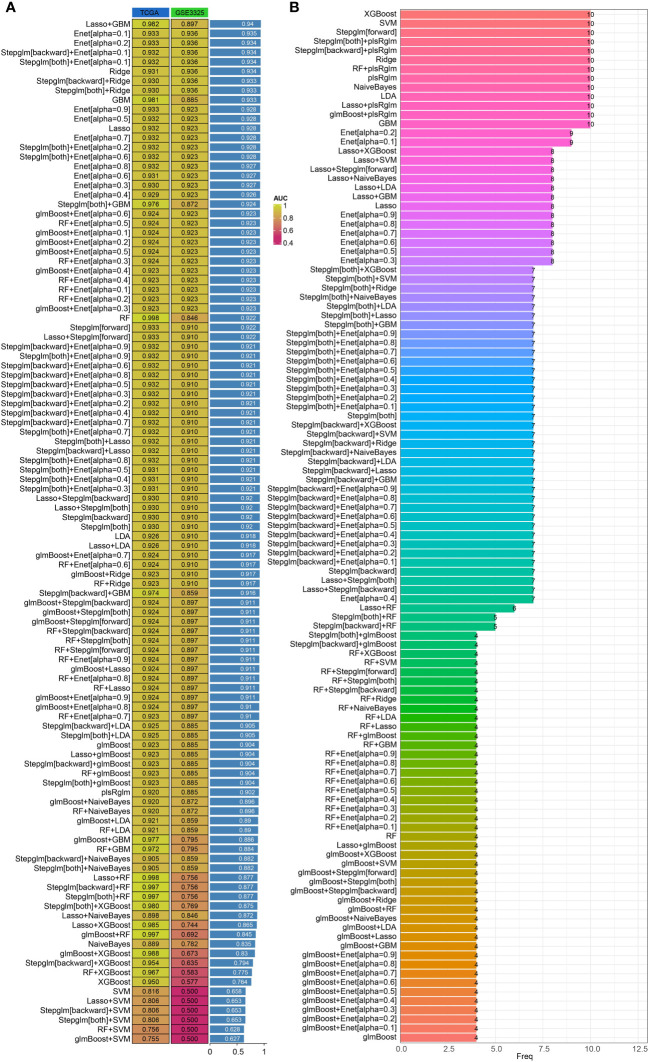
Construction of diagnostic models based on integrated machine learning models. **(A)** Predictive effectiveness of different algorithm combinations for PRAD diagnosis. **(B)** Number of genes incorporated by different combinations of algorithms.

Machine learning methods offer a convenient approach for identifying characteristic genes. In our study, we utilized multiple machine learning algorithms to create a diagnostic model related to angiogenesis. By analyzing the TCGA-PRAD and GSE3325 datasets, we used the expressions of FAM107A, C7orf31, TMEM100, GLIS1, QSOX1, KNDC1, MAP7D3, and ZNF536 in the TCGA-PRAD dataset as training data. Our findings revealed that both combinations of machine learning algorithms exhibited strong predictive capabilities for diagnosing PRAD patients in the training set. Subsequently, we validated the expressions of these genes in the GSE3325 dataset to confirm the effectiveness of our diagnostic model. In the verification set of GSE3325, only a few machine learning algorithm combinations showed poor results, while the majority achieved better predictions. Among these combinations, the LASSO+GBM algorithm combination stood out as the best diagnostic model, as it had the highest average AUC value (**Figure 3A**). Additionally, we presented the number of genes included in each algorithm combination for further clarity (**Figure 3B**).

### 3.4 Constructing prognostic models

**Figure 4:**
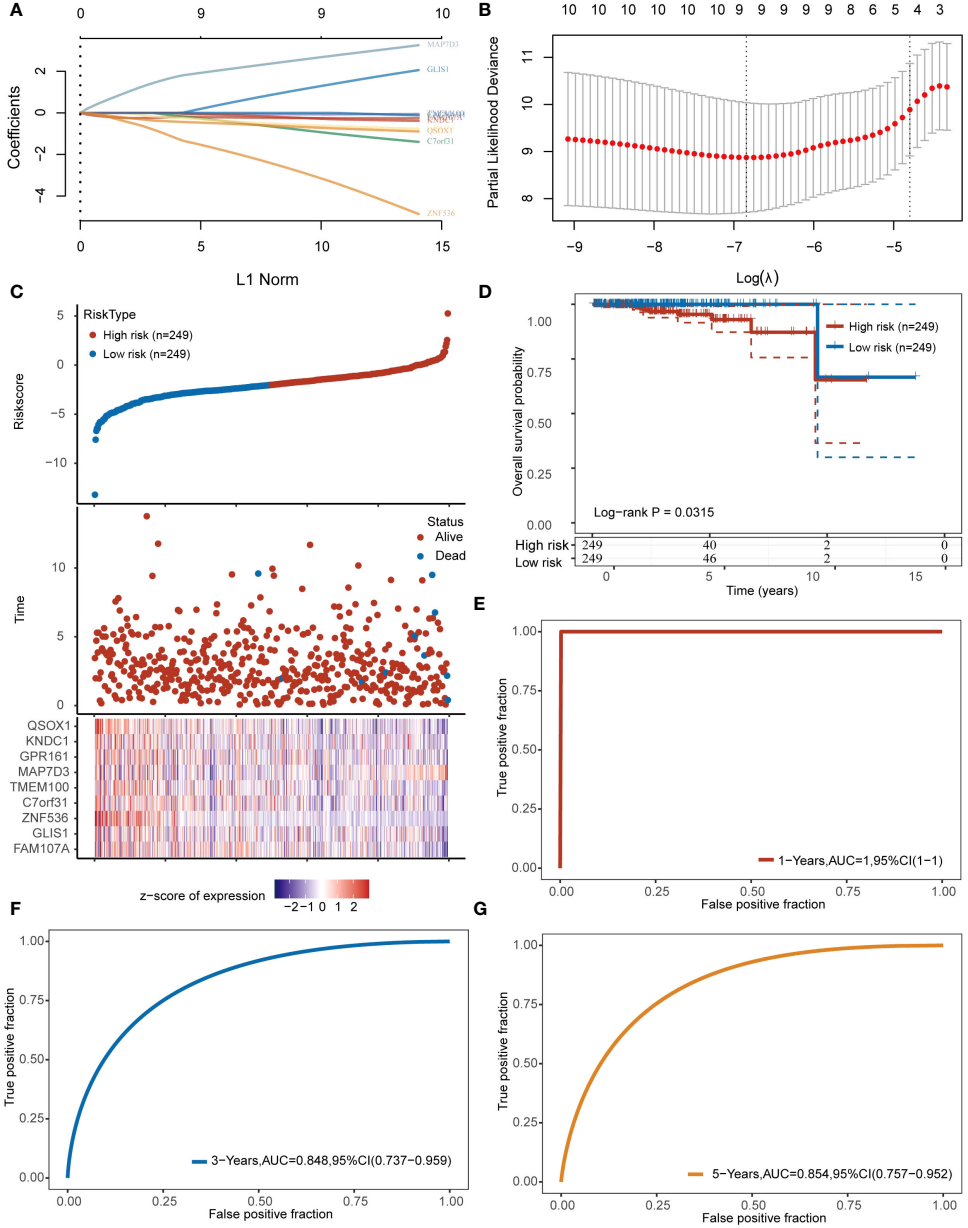
Constructing prognostic models based on angiogenesis genes. **(A, B)** 9 angiogenesis-related prognostic genes were included in the prognostic model. **(C)** The top represents the scatter plot of the Riskscore from low to high, the middle represents the scatter plot distribution of survival time and survival status corresponding to the Riskscore of different samples; the bottom represents the expression heat map of the genes included in the model. **(D)** Prognostic differences between high and low risk groups. **(E–G)** Prognostic modeling for predictive analysis of 1,3,5-year prognosis in patients with PRAD.

We examined 10 angiogenesis genes related to PRAD prognosis, including MAP7D3, FAMI07A, GLIS1, GPRI61, QSOX1, TMEM100, C7orf31, ZNF536, KNDC1, and CACNAIH, using the LASSO algorithm to develop a prognostic model. Subsequently, 9 genes, MAP7D3, FAMI07A, GLIS1, GPRI61, QSOX1, TMEM100, C7orf31, ZNF536, and KNDC1, were incorporated into the model (**Figures 4A, B**). Risk score= (-0.2524) *FAM107A+(1.237) *GLIS1+(-3.0503) *ZNF536+(-0.8946) *C7orf31+(-0.0732) *TMEM100+(2.6368) *MAP7D3+(-0.6038) *GPR161+(-0.2713) *KNDC1+(-0.7063) *QSOX1. The expression heatmap of these genes in PRAD samples was presented (**Figure 4C**). Using the gene expression data, the LASSO algorithm categorizes samples into high-risk and low-risk groups. Patients classified as high-risk typically experience a significantly poorer prognosis compared to those in the low-risk category (**Figure 4D**). Additionally, we evaluated the model’s predictive performance for 1-year, 3-year, and 5-year PRAD prognosis (**Figures 4E–G**). The ROC curve demonstrated strong predictive capability of the constructed prognostic model, with AUC values of 1, 0.848, and 0.854 for 1 year, 3 years, and 5 years, respectively.

### 3.5 Correlation analysis of prognostic models with PRAD immune infiltration and chemotherapeutic drug sensitivity

**Figure 5:**
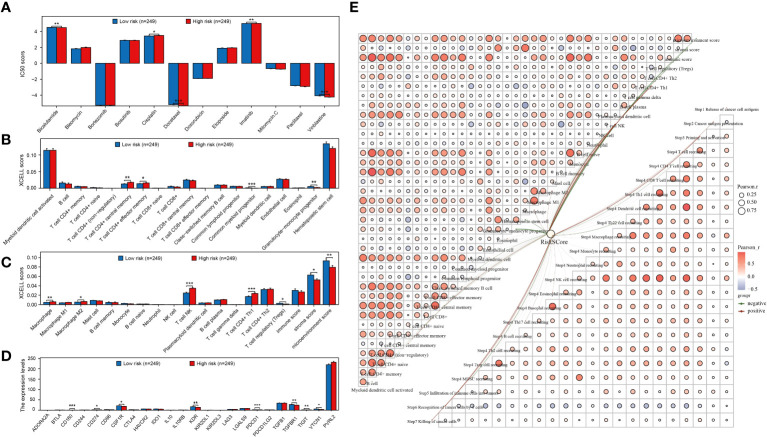
Prognostic models are strongly associated with PRAD chemotherapy and immunotherapy. **(A)** Analysis of the difference in IC50 scores of different chemotherapeutic drugs between high and low risk groups. **(B, C)** Analysis of immune cell infiltration levels between different groups. **(D)** Expression level analysis of different immunoinhibitors between high and low risk groups. **(E)** Network diagram of correlation between risk score and PRAD immune infiltration. *p < 0.05, **p < 0.01 and ***p < 0.001.

The study compared IC50 scores of various chemotherapy drugs in samples from high-risk and low-risk groups, revealing significant differences in 5 drugs between the groups (**Figure 5A**). Furthermore, the correlation between the constructed prognostic model and PRAD immune infiltration was examined, showing significant differences in the infiltration levels of 11 immune cells between high and low-risk groups (**Figures 5B, C**). Additionally, expression differences of immunosuppressants between the groups were analyzed, with 8immunoinhibitor-related genes showing significant differences (**Figure 5D**). Finally, the relationship between the prognostic model risk score and PRAD immune infiltration was explored using xCell and TIP methods, resulting in a correlation network diagram (**Figure 5E**).

### 3.6 MAP7D3 as the best prognostic gene among angiogenesis genes

**Figure 6:**
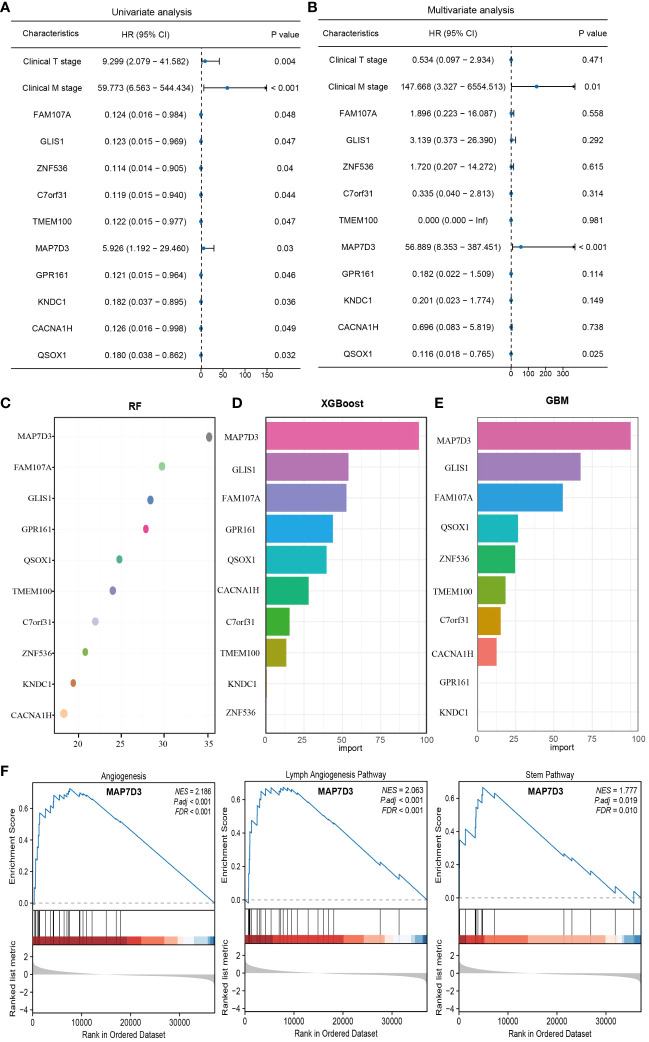
Multiple machine learning approaches to identify the best angiogenesis-related prognostic genes in PRAD. **(A)** Univariate COX regression analysis of prognostic differences in relevant indicators. **(B)** Multivariate COX regression to analyze prognostic differences in relevant indicators. **(C–E)** RF, XGBoost and GBM algorithms to screen prognostic genes. **(F)** Gene enrichment analysis based on MAP7D3 expression.

To delve deeper into identifying prognostic genes associated with angiogenesis, univariate and multivariate COX regression analyses were carried out on these genes along with clinically pertinent pathological factors (T stage, M stage). The findings from the multivariate COX regression analysis revealed that QSOX1, MAP7D3, and M stage could potentially function as prognostic indicators for patients with PRAD (**Figures 6A, B**). Subsequently, to identify the optimal prognostic biomarkers among these angiogenesis-related genes, we employed three machine learning methods: RF, XGBoost, and GBM. Combining the outcomes of multivariate COX regression analysis, we determined that MAP7D3 emerged as the most promising angiogenesis-related prognostic marker in PRAD (**Figures 6C–E**). Furthermore, gene enrichment analysis based on the high and low expression groups of MAP7D3 validated its association with PRAD angiogenesis. Interestingly, our findings also linked MAP7D3 to the stemness pathway (**Figure 6F**). The highly vascularized tumor microenvironment provides a conducive setting for the growth of these stem cells, perpetuating a detrimental cycle that contributes to tumor recurrence, metastasis, and drug resistance. Hence, we hypothesize that MAP7D3 may impact the prognosis of PRAD patients by modulating PRAD cell stemness and angiogenesis.

### 3.7 Analysis of MAP7D3 correlation with PRAD immunotherapy and chemotherapy

**Figure 7:**
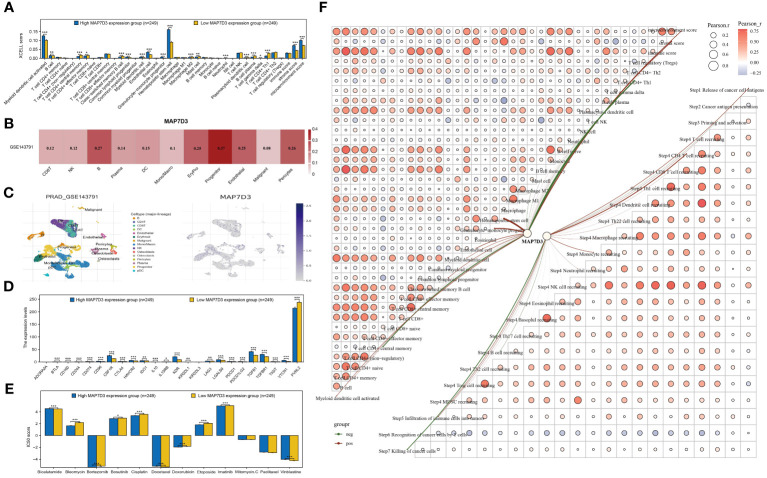
MAP7D3 was significantly associated with PRAD immunotherapy and chemotherapy. **(A)** Analysis of MAP7D3 correlation with PRAD immune cell infiltration based on XCELL algorithm. **(B, C)** Analysis of MAP7D3 correlation with PRAD immune cell infiltration based on single-cell dataset. **(D)** Analysis of MAP7D3 correlation with immunoinhibitor-related genes. **(E)** Analysis of the difference in IC50 scores of different chemotherapeutic drugs between high MAP7D3 expression and low MAP7D3 expression groups. **(F)** Network diagram of correlation between MAP7D3 expression and PRAD immune infiltration. *p < 0.05, **p < 0.01 and ***p < 0.001.

The XCELL algorithm was utilized to assess immune cell infiltration levels, unveiling notable variances in the infiltration of 19 immune cell types in PRAD samples categorized by high and low MAP7D3 expression (**Figure 7A**). Single-cell analysis aids in exploring gene expression patterns within individual cells and understanding intercellular signaling networks. Integrating clinical pathology data with scRNA-seq information from tumor samples has the potential to unveil novel diagnostic and prognostic biomarkers (28). Consequently, we investigated the immune infiltration of MAP7D3 through the TISCH2 database, and the findings indicated a significant association with B cells and progenitor cells (**Figures 7B, C**), aligning with the XCELL algorithm analysis results. Further examination of immunosuppressant-related gene expression in PRAD samples based on MAP7D3 expression levels revealed marked differences in the expression of 22 immunosuppressant-related genes (**Figure 7D**). Moreover, significant correlations were observed between MAP7D3 and the IC50 scores of 10 commonly used chemotherapy drugs (**Figure 7E**). Lastly, a correlation network graph depicting the relationship between MAP7D3 and immune cell infiltration levels was constructed using XCELL and TIP algorithms (**Figure 7F**).

### 3.8 Analysis of MAP7D3 correlation with angiogenesis-targeting drugs

**Figure 8:**
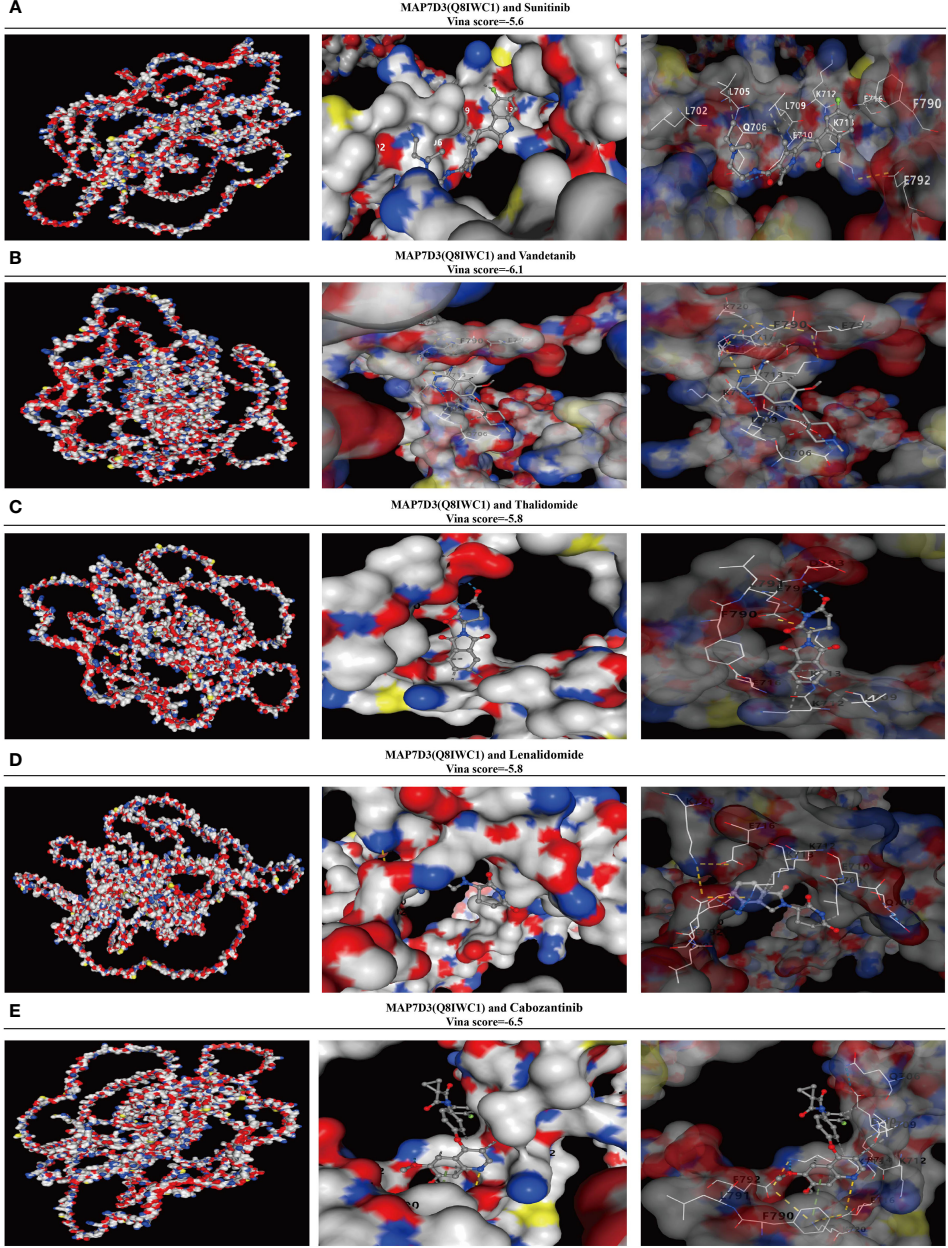
MAP7D3 is associated with angiogenesis drugs. **(A–E)** Molecular docking of MAP7D3 with the angiogenesis-targeting drugs sunitinib, Vandetanib, Thalidomide, Lenalidomide, and Cabozantinib.

In order to further investigate the potential of MAP7D3 as an angiogenesis-targeting drug, a correlation analysis was conducted at the molecular structure level comparing it with established angiogenesis-targeting drugs such as sunitinib, Vandetanib, Thalidomide, Lenalidomide, and Cabozantinib. The molecular structure of MAP7D3 was sourced from the AlphaFold website, while the 3D structures of the angiogenesis-targeting drugs were obtained from the PubChem website. The Vina score was utilized to assess the correlation between MAP7D3 and the other drugs, with a score of less than -5 generally indicating a strong binding activity. The findings revealed that MAP7D3 exhibited good binding activity with the selected angiogenesis-targeting drugs (**Figures 8A–E**).

### 3.9 Validation of MAP7D3 expression and prognostic value in PRAD

**Figure 9:**
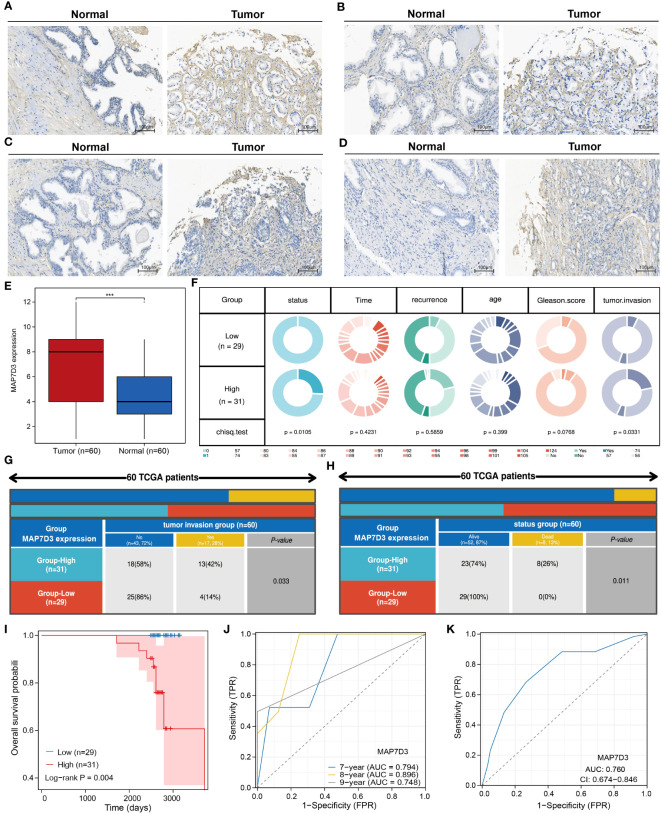
MAP7D3 is highly expressed in PRAD and may serve as a prognostic marker. **(A–E)** MAP7D3 expression in PRAD and corresponding normal tissues. **(F–H)** Correlation between MAP7D3 expression and different pathologic parameters in PRAD patients. **(I)** MAP7D3 expression and prognostic KM curves in PRAD patients. **(J)** Predictive ability of MAP7D3 expression for prognosis in PRAD patients. **(K)** Predictive ability of MAP7D3 expression for the diagnosis of PRAD patients. ***p < 0.001.

To ascertain the differential expression and prognostic significance of MAP7D3 in prostate adenocarcinoma (PRAD), we conducted immunohistochemistry experiments on 60 PRAD samples and their corresponding normal prostate tissue samples. Our analysis revealed that MAP7D3 was predominantly expressed in the cytoplasm of PRAD samples (**Figures 9A–D**). Comparing 60 cases of cancer with 60 adjacent cancer cases, we observed significantly higher MAP7D3 expression in the cancer samples (**Figure 9E**). Further categorizing the samples based on high and low MAP7D3 expression levels, we discovered a significant correlation between MAP7D3 expression, tumor invasion, and patient survival status (**Figure 9F**). Detailed examination of tumor invasion and patient survival status in the high and low MAP7D3 expression groups revealed distinct patterns (**Figures 9G, H**). Kaplan-Meier survival analysis demonstrated a notably worse prognosis for patients with high MAP7D3 expression compared to those with low expression. Additionally, receiver operating characteristic (ROC) curve analysis indicated that MAP7D3 could effectively predict 7-year, 8-year, and 9-year survival rates in PRAD patients (**Figures 9I, J**). Lastly, ROC curve analysis for the diagnostic potential of MAP7D3 in PRAD patients showed promising results, highlighting its utility in PRAD diagnosis (**Figure 9K**).

## 4 Discussion

Immune cells play a crucial role in tumor survival, as cancer cell metabolites and secretions from specific cells in the tumor microenvironment can impact immune cell activation, proliferation, differentiation, and overall function (29–31). The induction of immunosuppression and the ability to evade anti-tumor immune responses have been recognized as significant features in the progression of cancer, particularly through mechanisms such as tumor angiogenesis (32). With strong experimental evidence supporting tumor-dependent angiogenesis, researchers are increasingly focused on developing anti-angiogenic therapies (33). The approval of bevacizumab in 2004, the first FDA-approved anti-angiogenic drug, significantly improved progression-free survival in RCC patients undergoing combination chemotherapy (34). Subsequent studies have furthered anti-angiogenic strategies by proposing the normalization of tumor blood vessels to enhance drug and oxygen delivery. The use of angiogenesis inhibitors in cancer treatment, targeting the formation of new blood vessels in tumors, represents a promising approach for a variety of solid tumors (35). Nevertheless, obstacles like tumor regrowth, resistance to medication, absence of biomarkers, limited duration of effectiveness, and possible negative reactions continue to persist as a result of the intricate aspects of tumor vascularization and insufficient investigation. Even though existing medications that inhibit blood vessel formation may not be optimal for managing PRAD, an enhanced comprehension of the mechanisms driving PRAD vascularization could pave the way for the creation of superior tailored treatments for individuals with PRAD.

WGCNA is a bioinformatics algorithm utilized for extracting module information from high-throughput expression data, known for its efficiency and accuracy in biological data mining (36). In our study, we applied WGCNA to identify genes associated with angiogenesis in PRAD. By integrating differential analysis and prognostic assessment, we successfully pinpointed 10 angiogenesis-related genes with prognostic significance. Among these 10 angiogenesis-related genes, studies have reported that QSOX1, GLIS1 and FAM107A can be used as prognostic markers for PRAD patients (37–39). In recent years, the concept of precision medicine has emphasized subgroup typing of individual research subjects. Through consensus clustering analysis based on the expression of 10 angiogenesis-related genes, we divided the TCGA-PRAD samples into 2 clusters. Our findings revealed significant differences between these clusters, not only in prognosis but also in sensitivity to immunotherapy and chemotherapy drugs. To further analyze these differences, we conducted gene enrichment analysis and discovered that samples in cluster 2 were primarily associated with VEGF, PDGF, and PI3K/Akt signaling pathways. VEGF-A is a crucial regulator of angiogenesis, exerting a significant influence on tumor proliferation, metastasis and drug resistance. The key signaling pathway involved in both physiological and pathological angiogenesis is VEGF-A/VEGFR-2, which promotes various processes in endothelial cells and solid tumors. Platelet-derived growth factor (PDGF) serves as a primary stimulant for mesenchymal cell types like fibroblasts, smooth muscle cells, and glial cells, contributing to cell growth, wound healing, angiogenesis, and recruitment through paracrine or autocrine mechanisms. PDGF-BB, a well-studied factor in the PDGF family, not only enhances tissue fibrosis but also drives angiogenesis and drug resistance during tumor progression and anti-VEGF therapy (40–42). Additionally, the PI3K/Akt signaling pathway plays a significant role in tumor angiogenesis (43). These findings indirectly support the strong correlation between these 10 genes and angiogenesis. Machine learning is a prominent subject in current research. We utilized various machine learning methods to develop both a diagnostic and prognostic model for PRAD based on the expression of angiogenesis-related genes. Our model results consistently highlight the significant role of angiogenesis in the diagnosis and prognosis of PRAD. We validated the diagnostic model using GSE3325 and obtained satisfactory outcomes. However, due to limited availability of PRAD datasets with prognostic information, our prognostic model lacks validation. This limitation stems from missing gene expression data in the datasets used for model construction.

Multivariate COX regression analysis identified MAP7D3 and QSOX1 as prognostic biomarkers for PRAD among the 10 angiogenesis-related genes. High expression of QSOX1 has been linked to vascular invasion, neural invasion, prostate extension, increased pT stage, and higher pathological tumor stage in prostate cancer. These findings underscore the significant role of QSOX1 in PRAD (37). Subsequently, RF, XGBoost, and GBM machine learning methods were employed to identify the optimal prognostic genes, ultimately confirming MAP7D3 as the top prognostic gene associated with angiogenesis in PRAD. To uncover novel angiogenesis-related prognostic genes, we focused on MAP7D3 for further investigation. Single-cell RNA sequencing is an advanced genomics technology that enables comprehensive analysis of gene expression and genomic features at the single cell level, thereby facilitating in-depth study of cellular properties (44). Therefore, we not only used the XCELL algorithm, but also analyzed the correlation between MAP7D3 and immune cell infiltration in PRAD from the perspective of single cell analysis through the TISCH2 database. Analytical results demonstrated a close correlation between MAP7D3 expression and B cell and progenitor cell infiltration levels in PRAD samples. Furthermore, differences in IC50 scores of immunosuppressant-related genes and common chemotherapy drugs were observed between MAP7D3 high and low expression groups, suggesting a crucial role of MAP7D3 in PRAD immunotherapy and chemotherapy. Gene enrichment analysis indicated that MAP7D3 is not only linked to angiogenesis but also to stem cell pathways. Stem cells are precursor cells that have the ability to self-renew and differentiate into functionally mature, specialized cells in various human tissues Stem cells are precursor cells that have the ability to self-renew and differentiate into functionally mature special cells in various tissues of the human body (45). Importantly, evidence suggests that the interplay between tumor angiogenesis and cancer stem cells promotes tumor growth. Cancer stem cells can contribute to angiogenesis by releasing pro-angiogenic factors and differentiating into vascular endothelial cells, while tumor vasculature supports cancer stem cells (46, 47). This reciprocal interaction between tumor angiogenesis and stemness fuels tumor progression and metastasis. Therefore, the findings suggest that MAP7D3 may drive PRAD progression by regulating both angiogenesis and stem cells. Sunitinib, vandetanib, thalidomide, lenalidomide, and cabozantinib are currently utilized as angiogenesis-related targeted drugs in clinical settings. To investigate the potential of MAP7D3 as a target for angiogenic drug development, we conducted an analysis of its binding activity with these drugs using molecular docking. Our findings are promising, indicating that MAP7D3 exhibits strong binding activity with the aforementioned drugs, suggesting its potential as an angiogenesis drug target. Our study provides valuable insights into the significant role of angiogenesis in PRAD, drawing from various perspectives. However, it is important to note that the majority of our analyses were conducted using the TCGA-PRAD dataset. To strengthen the robustness of our findings, it is imperative to incorporate a larger sample size and diverse validation sets. Furthermore, conducting additional experiments to validate our conclusions is equally essential.

## 5 Conclusion

Our study employed a range of machine learning techniques to pinpoint 10 crucial angiogenesis-related genes in prostate cancer. Our findings validate the role of these genes in influencing PRAD immunotherapy, chemotherapy, and patient outcomes. Notably, both machine learning analysis and experimental validation underscore the significant prognostic impact of MAP7D3. Moreover, our research advocates for the potential of MAP7D3 as a promising target for the development of angiogenic drugs.

## Data availability statement

The datasets presented in this study can be found in online repositories. The names of the repository/repositories and accession number(s) can be found in the article/supplementary material.

## Ethics statement

The studies involving humans were approved by Ethics Committee of Shanghai Outdo Biotech Company. The studies were conducted in accordance with the local legislation and institutional requirements. The participants provided their written informed consent to participate in this study. Written informed consent was obtained from the individual(s) for the publication of any potentially identifiable images or data included in this article.

## Author contributions

YW: Conceptualization, Data curation, Formal analysis, Investigation, Writing – original draft. JH: Investigation, Software, Writing – original draft. QZ: Investigation, Methodology, Writing – original draft. JB: Supervision, Writing – original draft. YZ: Validation, Writing – original draft. HS: Methodology, Writing – original draft. BD: Funding acquisition, Writing – review & editing. MR: Funding acquisition, Visualization, Writing – review & editing.
